# First identification of marine diatoms with anti-tuberculosis activity

**DOI:** 10.1038/s41598-018-20611-x

**Published:** 2018-02-02

**Authors:** Chiara Lauritano, Jesús Martín, Mercedes de la Cruz, Fernando Reyes, Giovanna Romano, Adrianna Ianora

**Affiliations:** 1 Stazione Zoologica Anton Dohrn, Department of Integrative Marine Ecology, Naples, Italy; 2 Fundación MEDINA, Centro de Excelencia en Investigación de Medicamentos Innovadores en, Andalucía, Avda. del Conocimiento 34, Granada, 18016 Spain

## Abstract

Marine microalgae are considered a potentially new and valuable source of biologically active compounds for applications in several biotechnology sectors. They can be easily cultured, have short generation times and enable an environmentally-friendly approach to drug discovery by overcoming problems associated with the over-utilization of marine resources and the use of destructive collection practices. Considering the increasing rate of antibiotic-resistance bacteria and infections by fungi, 46 microalgae have been screened in this study for possible antibacterial and antifungal activities. Two different extraction methods have been used in order to increase the probability of finding positive hits. In particular, we screened microalgae in both control and nutrient stress conditions. We also tested different strains for 7 species in order to study potentially different bioactivities due to strain diversity. Results showed that extracts of two diatoms, *Skeletonema costatum* and *Chaetoceros pseudocurvisetus*, had anti-tuberculosis activity and were active only when cultured in the control and phosphate-starvation conditions, while the nitrogen starvation condition showed no activity. In addition, we tested both the organic and water extracts and found that only the organic extracts for both diatoms were active. The organic extracts of these two diatom species were not toxic on normal human cell lines.

## Introduction

Microalgae are eukaryotic plants that contribute up to 25% of global productivity and 50% of all aquatic productivity^1,2^. They are the basis of aquatic food webs and have colonized almost all biotopes, from temperate to extremes environments (e.g. cold/hot environments, hydrothermal vents). The number and diversity of microalgal species offer a great reservoir of compounds with possible applications in various biotechnology sectors (i.e. food, energy, health, environment and biomaterials)^3–8^.

Microalgae can be easily cultivated in photo-bioreactors (e.g. in 35,000 L bioreactors) to obtain huge biomass, have short generation times (doubling time = 5–8 h for some species) and represent a renewable and still poorly explored resource for drug discovery^4,9,10^. Their advantage is also their metabolic plasticity, dependent on their non-stressed or stressed status under different light, temperature and nutrient stress^11,12^. However, although a range of bioactivities have been observed from microalgal extracts, the active principles are often unknown^11–16^. Not much is known on the potential applications of microalgae as sources of anti-infective agents, but it has been suggested that they have evolved protection mechanisms against infections such as surface-fouling bacteria that are indigenous to ocean waters or because of competition for the same resources.

Extracts from different marine microalgae have shown the capability to inhibit bacterial growth with variable levels of activity. Lipophilic extracts of the diatom *Skeletonema costatum* showed significant antibacterial effects against *Listonella anguillarum*^17^, while extracts of the diatom *Phaeodactylum tricornutum* were active against both *L*. *anguillarum* (Gram−) and *Staphylococcus aureus* (Gram+)^18^. Kokou *et al*.^19^ screened extracts of the green algae *Tetraselmis chui* and *Nannochloropsis* sp. and the haptophyte *Isochrysis* sp. against six *Vibrio* bacterial strains and found that all the microalgae inhibited *Vibrio* growth. They also tested if light stress could change the antibacterial activity, but the activity was not influenced by the presence or absence of light. Sushanth and Rajashekhar^20^ tested the activity of extracts of the diatoms *Chaetoceros calcitrans* and *Skeletonema costatum*, and green alga *Nannochloropsis oceanica* against *S*. *aureus*, *Streptococcus pyogenes*, *Bacillus subtilis*, *E*. *coli*, *Proteus vulgaris*, *Klebsiella pneumoniae* and *Salmonella typhi*. All microalgal extracts were only active against *S*. *aureus*, *S*. *pyogenes* and *B*. *subtilis*. Rajendran *et al*.^21^ screened extracts of the green algae *Tetraselmis* sp. and *Dunaliella* sp. against seven different bacterial strains (i.e. *Bacillus* sp., *Escherichia coli*, *S*. *aureus*, *Salmonella* sp., *Pseudomonas aeruginosa*, *Klebsiella* sp. and *Enterococcus* sp.) and three fungal species (i.e. *Rhizopus* sp., *Anidubans* sp. and *Fusarium* sp.). *Tetraselmis* sp. showed antibacterial activity against *Pseudomonas* sp., *Enterococcus* sp. and *E*. *coli* and antifungal activity against all the three fungal strains, while *Dunaliella* sp. showed activity against *Rhizopus* sp. and *Fusarium* sp. Pane *et al*.^22^ also analyzed the bioactivity of *Dunaliella* extracts: they tested the ability of *D*. *salina* to inhibit the growth of 114 bacterial and 11 fungal strains isolated from patients with external otitis and found interesting activities especially against *S*. *aureus*, *P*. *aeruginosa*, *E*. *coli* and *Klebsiella* spp.

Ingebrigtsen *et al*.^11^ and Lauritano *et al*.^12^ tested the antibacterial properties of several microalgae cultured in both control and stressful conditions. Ingebrigtsen *et al*.^11^ tested the less polar fraction of five pelagic North Atlantic marine diatoms (i.e. *Attheya longicornis*, *Chaetoceros socialis*, *Chaetoceros furcellatus*, *Skeletonema marinoi* and *Porosira glacialis*) grown in 4 different light/temperature conditions against methicillin resistant *S*. *aureus* (MRSA), *Enterococcus faecalis*, *S*. *aureus*, *P*. *aeruginosa* and *E*. *coli*). Only the diatom *A*. *longicornis* was active against bacteria MRSA and *S*. *aureus*, and had a weak activity against *E*. *faecalis*. In addition, only *A*. *longicornis* cultures exposed to high light and high temperature had this activity. They did not observe positive hits against *P*. *aeruginosa* and *E*. *coli*. Lauritano *et al*.^12^ screened crude extracts of 32 microalgal species (21 diatoms, 7 dinoflagellates and 4 flagellates; strains collected mainly in the Mediterranean Sea as reported in Table 1) grown in three different culturing conditions, i.e. replete medium, nitrogen- and phosphate-starved media against *E*. *coli*, *P*. *aeruginosa*, *S*. *aureus*, *E*. *faecalis* and *Streptococcus B*. No apparent bioactivity against any of the tested strains was observed, except for two strains of the diatom *Skeletonema marinoi* (i.e. FE60 and FE6) that inhibited *S*. *aureus* growth by 97 and 96%, respectively. Interestingly, these strains were active only in stressful conditions: strain FE60 was active only when cultured in nitrogen-starved medium, while strain FE6 in the phosphate-starved medium. Altogether, the results of Ingebrigtsen *et al*.^11^ and Lauritano *et al*.^12^ confirmed that culturing conditions are very important in triggering the production of the bioactive molecules of interest and also testify that bioactivity is strain-dependent. The only study, to our knowledge, that tested activity against *Mycobacterium tuberculosis* was performed by Prakash *et al*.^23^ that found interesting activity in extracts of *Isochrysis galbana*.

**Table 1:** reports the Stazione Zoologica culture collection code, species name, medium (M) used for the culturing, class, sampling location (Samp. Location) and cell concentrations used for the chemical extractions of the microalgae cultured in control, nitrogen- and phosphate-starvation (C, N and P, respectively).

Code	Species name	M	Samp. Location	Class	Cell conc in C, N and P
FE10	*Skeletonema marinoi*	F/2	Mediterranean Sea	Coscinodiscophyceae	10^6^; 10^5^; 10^5^;
FE107*	*Alexandrium tamutum*	K	Mediterranean Sea	Dinophyceae	10^4^; 10^3^; 10^3^;
FE108*	*Alexandrium andersonii*	K	Mediterranean Sea	Dinophyceae	10^4^; 10^3^; 10^3^;
FE109^a^	*Alexandrium andersonii*	K	Mediterranean Sea	Dinophyceae	10^4^; 10^3^; 10^3^;
FE114	*Scrippsiella trochoidea*	K	Mediterranean Sea	Dinophyceae	10^4^; 10^3^; 10^3^;
FE119*	*Ostreopsis ovata*	K	Mediterranean Sea	Dinophyceae	10^4^; 10^3^; 10^3^;
FE126*	*Alexandrium minutum*	K	Mediterranean Sea	Dinophyceae	10^5^; 10^4^; 10^4^;
FE202*	*Rhodomonas baltica*	F/2	Mediterranean Sea	Cryptophyceae	10^5^; 10^4^; 10^4^;
FE208*^b^	*Rhinomonas reticulata*	F/2	Mediterranean Sea	Cryptophyceae	10^5^; 10^4^; 10^4^;
FE25*	*Skeletonema costatum*	F/2	Atlantic Ocean	Coscinodiscophyceae	10^6^; 10^5^; 10^5^;
FE3^c^	*Skeletonema costatum*	F/2	Atlantic Ocean	Coscinodiscophyceae	10^6^; 10^5^; 10^5^;
FE300*	*Coscinodiscus actinocyclus*	F/2	Mediterranean Sea	Bacillariophyceae	10^6^; 10^5^; 10^5^;
FE315*	*Lauderia annulata*	F/2	Mediterranean Sea	Coscinodiscophyceae	10^6^; 10^5^; 10^5^;
FE321*	*Nitzschia closterium*	F/2	Mediterranean Sea	Bacillariophyceae	10^6^; 10^5^; 10^5^;
FE322*	*Leptocylindrus danicus*	F/2	Mediterranean Sea	Coscinodiscophyceae	10^5^; 10^4^; 10^4^;
FE324*	*Chaetoceros affinis*	F/2	Mediterranean Sea	Coscinodiscophyceae	10^5^; 10^4^; 10^4^;
FE325	*Asterionellopsis glacialis*	F/2	Mediterranean Sea	Fragilariophyceae	10^6^; 10^5^; 10^5^;
FE326*	*Odontella mobiliensis*	F/2	Mediterranean Sea	Coscinodiscophyceae	10^6^; 10^5^; 10^5^;
FE327	*Chaetoceros socialis*	F/2	Mediterranean Sea	Coscinodiscophyceae	10^5^; 10^4^; 10^4^;
FE330	*Chaetoceros curvisetus*	F/2	Mediterranean Sea	Coscinodiscophyceae	10^5^; 10^4^; 10^4^;
FE330A	*Chaetoceros curvisetus*	F/2	Mediterranean Sea	Coscinodiscophyceae	10^5^; 10^4^; 10^4^;
FE331	*Chaetoceros pseudocurvisetus*	F/2	Mediterranean Sea	Coscinodiscophyceae	10^5^; 10^4^; 10^4^;
FE340	*Thalassiosira rotula*	F/2	Mediterranean Sea	Coscinodiscophyceae	10^6^; 10^5^; 10^5^;
FE4*	*Thalassiosira rotula*	F/2	Mediterranean Sea	Coscinodiscophyceae	10^6^; 10^5^; 10^5^;
FE40A	*Skeletonema marinoi*	F/2	Mediterranean Sea	Coscinodiscophyceae	10^6^; 10^5^; 10^5^;
FE41A	*Skeletonema marinoi*	F/2	Mediterranean Sea	Coscinodiscophyceae	10^6^; 10^5^; 10^5^;
FE41B	*Skeletonema marinoi*	F/2	Mediterranean Sea	Coscinodiscophyceae	10^6^; 10^5^; 10^5^;
FE42A	*Skeletonema marinoi*	F/2	Mediterranean Sea	Coscinodiscophyceae	10^6^; 10^5^; 10^5^;
FE43	*Skeletonema marinoi*	F/2	Mediterranean Sea	Coscinodiscophyceae	10^6^; 10^5^; 10^5^;
FE44A	*Skeletonema marinoi*	F/2	Mediterranean Sea	Coscinodiscophyceae	10^6^; 10^5^; 10^5^;
FE44B	*Skeletonema marinoi*	F/2	Mediterranean Sea	Coscinodiscophyceae	10^6^; 10^5^; 10^5^;
FE46	*Skeletonema marinoi*	F/2	Mediterranean Sea	Coscinodiscophyceae	10^6^; 10^5^; 10^5^;
FE5*	*Thalassiosira weissflogii*	F/2	Atlantic Ocean	Coscinodiscophyceae	10^6^; 10^5^; 10^5^;
FE76A	*Skeletonema tropicum*	F/2	Mediterranean Sea	Coscinodiscophyceae	10^6^; 10^5^; 10^5^;
FE80*	*Thalassiosira rotula*	F/2	Mediterranean Sea	Coscinodiscophyceae	10^6^; 10^5^; 10^5^;
FE80A	*Thalassiosira rotula*	F/2	Mediterranean Sea	Coscinodiscophyceae	10^6^; 10^5^; 10^5^;
FE85*^d^	*Skeletonema costatum*	F/2	Atlantic Ocean	Coscinodiscophyceae	10^6^; 10^5^; 10^5^;
FE88^e^	*Asterionellopsis glacialis*	F/2	Atlantic Ocean	Fragilariophyceae	10^6^; 10^5^; 10^5^;
FE92^f^	*Chaetoceros socialis*	F/2	Mediterranean Sea	Coscinodiscophyceae	10^5^; 10^4^; 10^4^;
FE94^g^	*Phaeodactylum tricornutum*	F/2	North Atlantic	Bacillariophyceae	10^6^; 10^5^; 10^5^;
L1*	*Bacteriastrum hyalinum*	F/2	Mediterranean Sea	Coscinodiscophyceae	10^5^; 10^4^; 10^4^;
L2*	*Guinardia striata*	F/2	Mediterranean Sea	Coscinodiscophyceae	10^5^; 10^4^; 10^4^;
L3*	*Lepidodinium viride*	K	Mediterranean Sea	Dinophyceae	10^4^; 10^3^; 10^3^;
L4*	*Proboscia alata*	F/2	Mediterranean Sea	Coscinodiscophyceae	10^5^; 10^4^; 10^4^;
MC1098_2*	*Pseudo-nitzschia pseudodelicatissima*	F/2	Mediterranean Sea	Bacillariophyceae	10^6^; 10^5^; 10^5^;
MC1098_3*	*Prorocentrum gracile*	K	Mediterranean Sea	Dinophyceae	10^5^; 10^4^; 10^4^;

*These species have been previously tested against *E*. *coli* and MRSA in Lauritano *et al*.^12^. ^a^Species from Bigelow National Center for Marine Algae and Microbiota named CCMP2222. ^b^Species from Culture collection of algae and protozoa named CCAP995/2. ^c^Species from Bigelow National Center for Marine Algae and Microbiota named CCMP780. ^d^Species from Roscoff Culture Collection named RCC1716. ^e^Species from Roscoff Culture Collection named RCC1712. ^f^Species from Bigelow National Center for Marine Algae and Microbiota named CCMP3263. ^g^Species from Bigelow National Center for Marine Algae and Microbiota named CCMP2561.

Some studies also showed the capacity of certain microalgae to have antifungal activity^24,25^. Sushanth and Rajashekhar^20^ tested the activity of extracts of *Chaetoceros calcitrans*, *Skeletonema costatum* and *Nannochloropsis oceanica* against *Candida albicans*, *Fusarium moniliforme*, *Aspergillus flavus* and *Aspergillus niger*. Only *S*. *costatum* showed antifungal activity and, in particular, against *F*. *moniliforme*. Nuzzo *et al*.^25^ reported the activity of extracts of the dinoflagellate *Amphidinium carterae* to inhibit the growth of the fungus *C*. *albicans*. In some cases, antimicrobial activity was associated with general cytotoxicity, limiting their use as potential drugs in human therapy^26^.

The aim of this study was to screen 46 microalgae (Table 1) for possible antibacterial and antifungal activities. In particular, we screened 36 diatoms, 8 dinoflagellates and 2 flagellates for possible growth inhibition activities against the Gram-negative bacterium *Escherichia coli*, the Gram-positive bacteria methicillin resistant *Staphylococcus aureus*, *Mycobacterium tuberculosis* and *Mycobacterium bovis*, and the fungus *Aspergillus fumigatus*. The microalgae used in this study have been tested both when cultured in control conditions (control medium without any stress) and in nutrient-starvation stress exposure (i.e. nitrogen- and phosphate-starvation) conditions. In addition, when possible, different strains of the same species were screened in order to assess both strain- and stress-dependent bioactivities. In particular, two strains of *Alexandrium andersonii*, two of *Asterionellopsis glacialis*, two of *Chaetoceros curvisetus*, two of *Chaetoceros socialis*, three of *Skeletonema costatum*, five of *Thalassiosira rotula* and nine of *Skeletonema marinoi* were analysed. In order to increase the probability to find positive hits, 2 different extraction methods were used in this study. The first method implied the use of the resin Amberlite XAD16N (Sigma- Aldrich) that is a macroreticular, styrene-divinyl benzene copolymer, non ionic resin that adsorbs and releases ionic species through hydrophobic and polar interactions. The second method was based on liquid extraction with acetone and dichloromethane (See methods for details).

The constant appearance and evolution of new antibiotic resistant organisms are the strong motivation for the search for new bioactive microalgal extracts, especially against *M*. *tuberculosis* and *M*. *bovis* that have been very poorly studied. *M*. *tuberculosis* is one of the most lethal infectious pathogens known. It infects about one third of the world’s population. In 2014, it caused 9.6 million cases of tuberculosis and killed 1.5 million people worldwide, while, in 2015, it caused 1.4 million deaths. The six countries with the largest number of incident cases (60%) in 2015 were India, Indonesia, China, Nigeria, Pakistan, and South Africa^27^. *M*. *bovis* is the causative agent of tuberculosis in cattle but can jump the species barrier and cause tuberculosis in humans and other mammals too^28^. The aim of this study was therefore to give a broad overview of microalgal anti-infective activities, including screenings against commonly studied or poorly studied bacteria/fungi, the testing of different strains and stressful culturing conditions and including LC/MS dereplication of bioactive extracts.

## Materials and Methods

### Microalgae culturing and maintenance

Microalgae (46 species) were selected from the Stazione Zoologica Anton Dohrn culture collection for culturing and antimicrobial activity screening (Table 1). 36 diatoms, 8 dinoflagellates and 2 flagellates were selected from those that have previously been shown to have anti-grazing and anti-proliferative activities on their predators at sea^29–31^ or species responsible for toxic blooms worldwide^32,33^. Species were previously identified by light microscopy and 18S or 28S sequencing. Diatoms and flagellates were grown in Guillard’s f/2 medium^34^ (For flagellates f/2 without silicates) and dinoflagellates in Keller medium^35^ in 10 L polycarbonate bottles. As for Lauritano *et al*.^12^, species were grown in control, nitrogen- and/or phosphate- starved media (90 μM NO^3−^ for N-starved and 0.5 µM PO_4_^2−^ for P-starved media). Species code with /2 indicate N-starved condition, while /3 P-starved. Cultures were kept in a climate chamber at 19 °C at 100 µmol photons m^−2^ s^−1^ at a 12:12 h light:dark cycle. Initial cell concentrations were about 5,000 cells/mL for each experiment and at the end of the stationary phase, cultures were centrifuged for 30 min at 4 °C at 3000 g and pellets (for the approximate cell concentration used for the chemical extractions of microalgae cultured in control, nitrogen- and phosphate-starvation see Table 1) kept at −80 °C until chemical extraction.

### Chemical extraction

Chemical extraction was performed with two different methods. The first method (named Method 1) included the use of the resin Amberlite XAD16N (20–60 mesh, Sigma-Aldrich, St. Louis, MO). In particular, 50 mL of distilled water was added to the microalgal pellets and samples were sonicated at 30 kHz for 30 s twice by using the Branson Sonifier 250. The same volume of acetone was added and, after 50 min mixing at room temperature, samples were evaporated under nitrogen stream down to half of their volume. About 1 g of Amberlite XAD16N resin (20–60 mesh, Sigma-Aldrich) was added to each sample. Amberlite® XAD16N is a macroreticular, styrene-divinyl benzene copolymer, non ionic resin that adsorbs and releases ionic species through hydrophobic and polar interactions. In addition, the resin favours the extraction of hydrophobic compounds up to 40,000 MW (http://www.sigmaaldrich.com/catalog/product/sigma/xad16?lang = it&region = IT). After 50 min of mixing at room temperature, samples were centrifuged (15 min at 3500 g at room temperature) and 18 mL of water were added to the resin for a washing step. After 50 min of mixing at room temperature, a centrifugation step (15 min at 3500 g at room temperature) allowed the elimination of water and the resin was incubated with 10 mL acetone for 50 min. Centrifugation (at 3500 g) for 15 min at room temperature allowed the resin to settle and the supernatants, that were the final extracts, were freeze-dried and stored at −20 °C until screening.

The second extraction method (named Method 2) was performed as in D’Ippolito *et al*.^36^. In particular, 2 mL of distilled water per each g of fresh sample weight were added to each microalgal pellets. Briefly, samples were sonicated at 30 kHz for 30 s twice by using the Branson Sonifier 250. A volume of acetone was added and samples were centrifuged at 3600 rpm for 6 min at 4 °C. The supernatant was transferred in clean tubes and stored on ice. Water and acetone were added again to the remaining pellets and the centrifugation step was repeated for two other times. A volume of dichloromethane was added to the recovered supernatant, mixed and centrifuged at 3600 rpm for 6′ at 15 °C. The organic phase was stored on ice, while the extraction with dichloromethane was repeated two times. The recovered water was freeze-dried and stored as water samples. On the contrary, the organic samples were pooled and treated with anhydrous sodium sulfate in order to remove the residues of water and dried under vacuum. Both water and organic samples for each species cultured in control and phosphate starvation conditions were stored at −20 °C until screening.

### Cytotoxicity Assay

Cytotoxicity was evaluated after 24 h exposure in human hepatocellular liver carcinoma (HepG2, ATCC HB-8065™) cells as in Lauritano *et al*.^12^. Briefly, 20,000 HepG2 cells were seeded per well, grown overnight, and then incubated with 50 μg/mL test extract diluted in MEM Earle’s supplemented with gentamycin (10 μg/mL), non-essential amino acids (1%), sodium pyruvate (1 mM), L-alanyl-L-glutamine (2 mM), but without FBS (total volume was 100 µl). Ten μL of CellTiter 96® AQueous One Solution Reagent (Promega, Madison, WI, USA) was added and plates were then further incubated for 1 h. Absorbance was measured at 485 nm in a DTX 880 Multimode Detector. Results were calculated as % survival compared to negative (assay media) and positive (Triton X-100; Sigma-Aldrich) controls. The screening was performed using 3 biological replicates and 9 technical replicates.

### Antibacterial Assay

The Gram-negative bacterium *Escherichia coli* (MB2884) and the Gram-positive bacterium methicillin resistant *Staphylococcus aureus* (MB5393) were used as test organisms and antibacterial tests were performed as in Audoin *et al*.^37^. Briefly, for the liquid media antibacterial tests, thawed stock inocula suspensions from cryovials of each microorganism (MRSA and *E*. *coli*) were streaked onto Luria-Bertani agar plates (LBA, 40 g/L) and incubated at 37 °C overnight to obtain isolated colonies. Single colonies of each microorganism were inoculated into 10 mL of Luria-Bertani broth medium (LB, 25 g/L in 250 mL Erlenmeyer flasks) and incubated overnight at 37 °C with shaking at 220 rpm and then diluted in order to obtain assay inocula of approximately 1.1 × 10^6^ CFU/mL (MRSA) or 5–6 × 10^5^ CFU/mL (*E*. *coli*). For the assay, 90 μL/well of the diluted inoculum were mixed with 800 μg/mL/well of each microalgal organic extract and LB medium. For the two active microalgae, a serial dilution screening was performed (800 μg/mL, 200 μg/mL and 128 μg/mL). In addition, for these microalgae also the water extract was tested in order to identify possible differences between organic and water extracts. Kanamycin and amphotericin B (MRSA) and novobiocin and amphotericin B (*E*. *coli*) were included as internal plate controls. Absorbance was measured at 612 nm with a Tecan Ultra Evolution spectrophotometer (Tecan, Durham, USA) at T0 (zero time) and immediately after that, plates were statically incubated at 37 °C for 20 h. After this period, the assay plates were shaken using the DPC Micromix-5 and once more the absorbance at OD612 nm was measured at Tf (final time). The screening was performed using 3 biological replicates and 9 technical replicates.

### Anti-TB Assay

The anti-tubercular activity of the extracts was determined using the REMA method^38^ *M*. *tuberculosis* H37Ra ATCC 25177 and *M*. *bovis* ATCC 35734 were grown for 15–21 days in Middlebrook 7H9 broth (Becton Dickinson ref 271310) supplemented with 10% ADC enrichment (Becton Dickinson ref. 211887) containing albumin, dextrose, and catalase; 0.5% glycerol as a carbon source; and 0.25% Tween 20 to prevent clumping. Suspensions were prepared, and the turbidity was adjusted to 0.5 OD at 600 nm. Then, further dilutions were made to reach the final bacterial suspension concentration of 5 × 10^5^ CFU/mL for the assay. A volume of 90 μL of the inoculum was added to each well of a 96-well microtiter plate containing the 10 μL of extracts (20% DMSO). Growth controls containing no antibiotic and sterility controls without inoculation were also included. Streptomycin was used as positive control. Plates were incubated for 7 days at 5% CO_2_ 95% humidity and 37 °C. The assays were set up in triplicate.

After this incubation, 30 μL of 0.02% resazurin and 15 μL of Tween 20 were added to each well, incubated 24 hours and assessed for color development. A change from blue to pink indicates reduction of resazurin and therefore bacterial growth. The wells were read for color change and the data were quantified by measuring fluorescence (excitation 570 nm, emission 615 nm) using a VICTOR multilabel counter (Perkin Elmer, Waltham, MA). The MIC (minimum inhibitory concentration) was defined as the lowest concentration resulting in a 90% growth inhibition of microorganism. For the Resazurin Solution, Resazurin sodium salt (C_12_H_6_NO_4_Na; R7017, Sigma-Aldrich) stock solution of 0.02 g was dissolved in 100 mL of sterile distilled water and sterilized by filtration.

### Analysis of compounds using LC-MS

Samples were analyzed by HPLC-UV-HRMS on an Agilent 1200 RR coupled to a Bruker maXis time of flight spectrometer with electrospray ionization as previously described^39^. Dereplication of extract components was performed using the procedures, software (MS Gold) and databases (Fundación MEDINA reference library and the Chapman&Hall dictionary of natural products) described in Pérez-Victoria *et al*.^40^.

### Statistical analysis

Statistical significances for all the assays performed in this study were determined by Student’s t-test using GraphPad Prim statistic software, V4.00 (GraphPad Software, San Diego, California, USA). Data were considered significant when at least p was < 0.05 (* for p < 0.05, ** for p < 0.01 and *** for p < 0.001).

## Results and Discussion

### Bioactivity results

**Figure 1:**
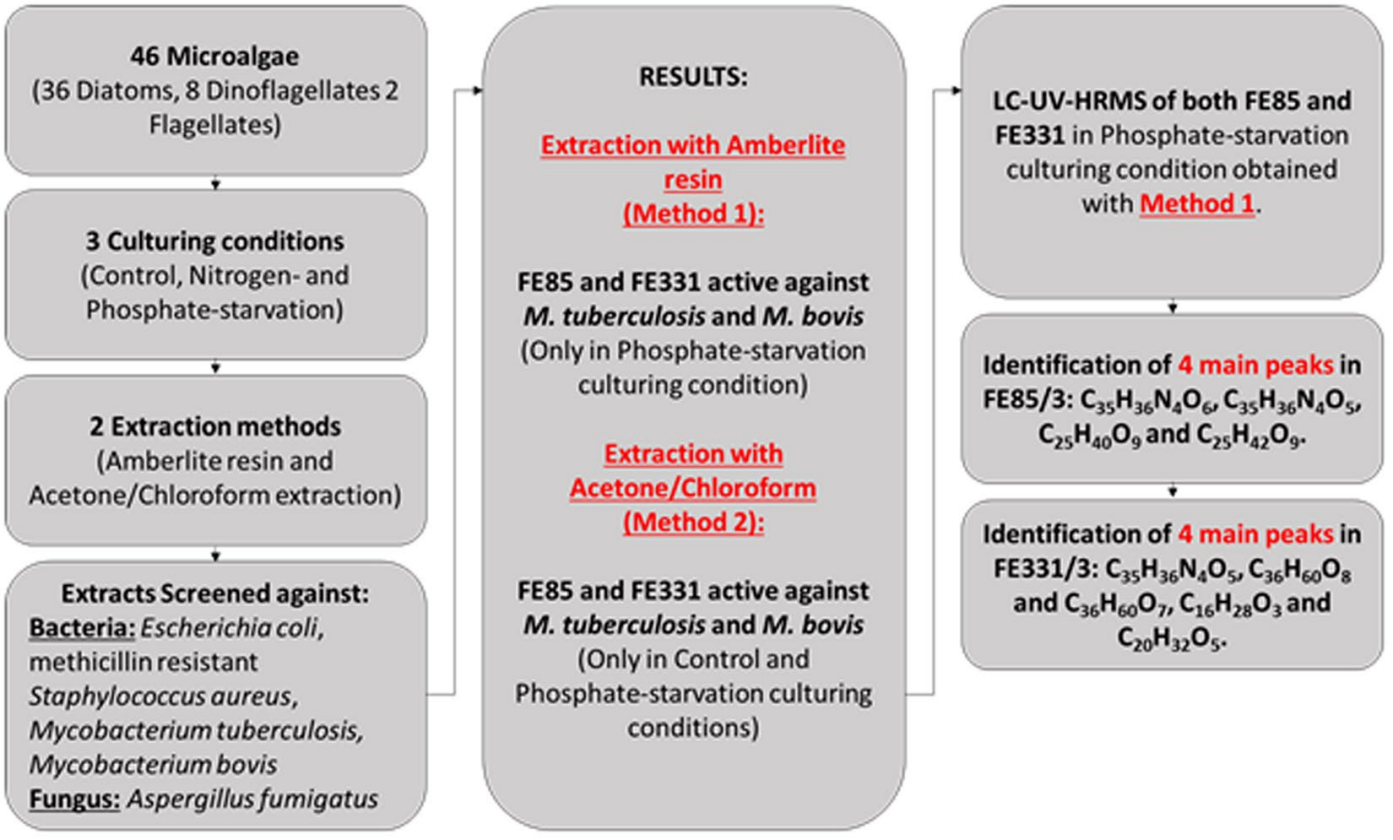
Flowchart of the experimental procedure. 46 microalgae (36 diatoms, 8 dinoflagellates and 2 flagellates) were cultivated in 3 culturing conditions. Microalgal pellets were extracted with 2 methods and screened against several bacteria and a fungus. Two diatoms showed activity and dereplication was performed in order to identify the main peaks.

Of the microalgae screened in this study, two diatoms showed a strong anti-tuberculosis activity, *Skeletonema costatum* FE85 and *Chaetoceros pseudocurvisetus* FE331 (Fig. 1 shows the flowchart of the experimental procedure). Extracts of *Skeletonema costatum* have previously been shown to have activity against *Listonella anguillarum*^17^, *S*. *aureus*, *S*. *pyogenes* and *B*. *subtilis*^20^. Lauritano *et al*.^12^ screened the same *S*. *costatum* strains of this study (i.e. FE85 and FE25) against *E*. *coli*, *P*. *aeruginosa*, *S*. *aureus*, *E*. *faecalis* and *Streptococcus B*, but did not find any activity. To our knowledge, extracts of the second diatom, *Chaetoceros pseudocurvisetus*, have never been screened for possible antimicrobial activities.

**Figure 2:**
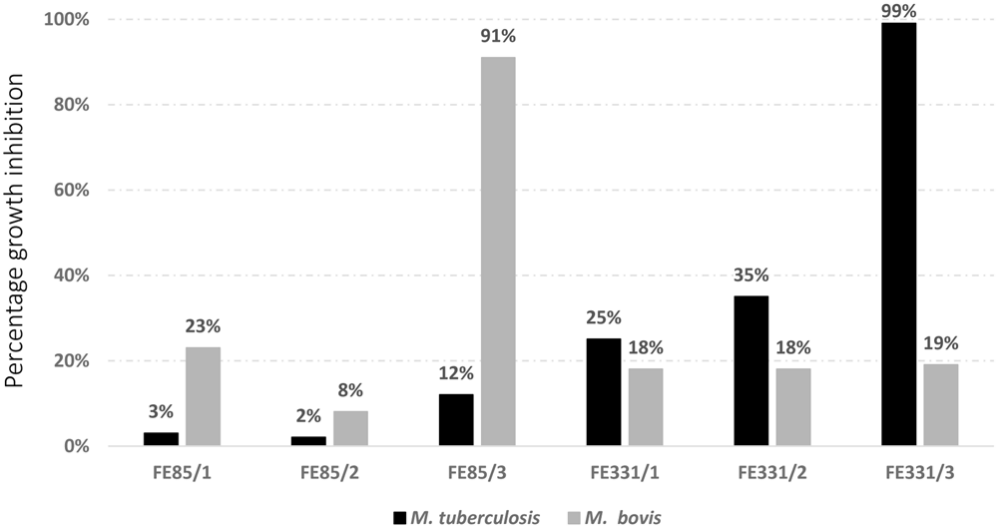
Percentage inhibition activity of *Skeletonema costatum* and *Chaetoceros pseudocurvisetus* by using the Amberlite resin extraction method. Figure 2 reports the percentage inhibition activity of *Skeletonema costatum* FE85 and *Chaetoceros pseudocurvisetus* FE331 cultured in control, nitrogen- and phosphate-starvation conditions (/1, /2 and /3, respectively) against *Mycobacterium tuberculosis* and *Mycobacterium bovis*. Extracts were obtained by using the Amberlite resin.

Chemical extraction was performed with two different methods in order to evaluate if possible antimicrobial activities may be related to the extraction methods used and to increase the probability to extract an active compound. Regarding the extracts obtained with the extraction Method 1 (i.e. with Amberlite resin), both FE85 and FE331 were active only when cultured in the phosphate-starvation conditions (FE85/3 and FE331/3; Fig. 2). In particular, 800 μg/mL extracts of FE85/3 had 91% growth inhibition against *M*. *bovis* (Student’s t-test, p < 0.001), while 800 μg/mL extracts of FE331/3 showed a 99% inhibition activity against *M*. *tuberculosis* (Student’s t-test, p < 0.001). The other culturing conditions showed between 2 and 35% inhibition (See Fig. 2 for details).

**Figure 3:**
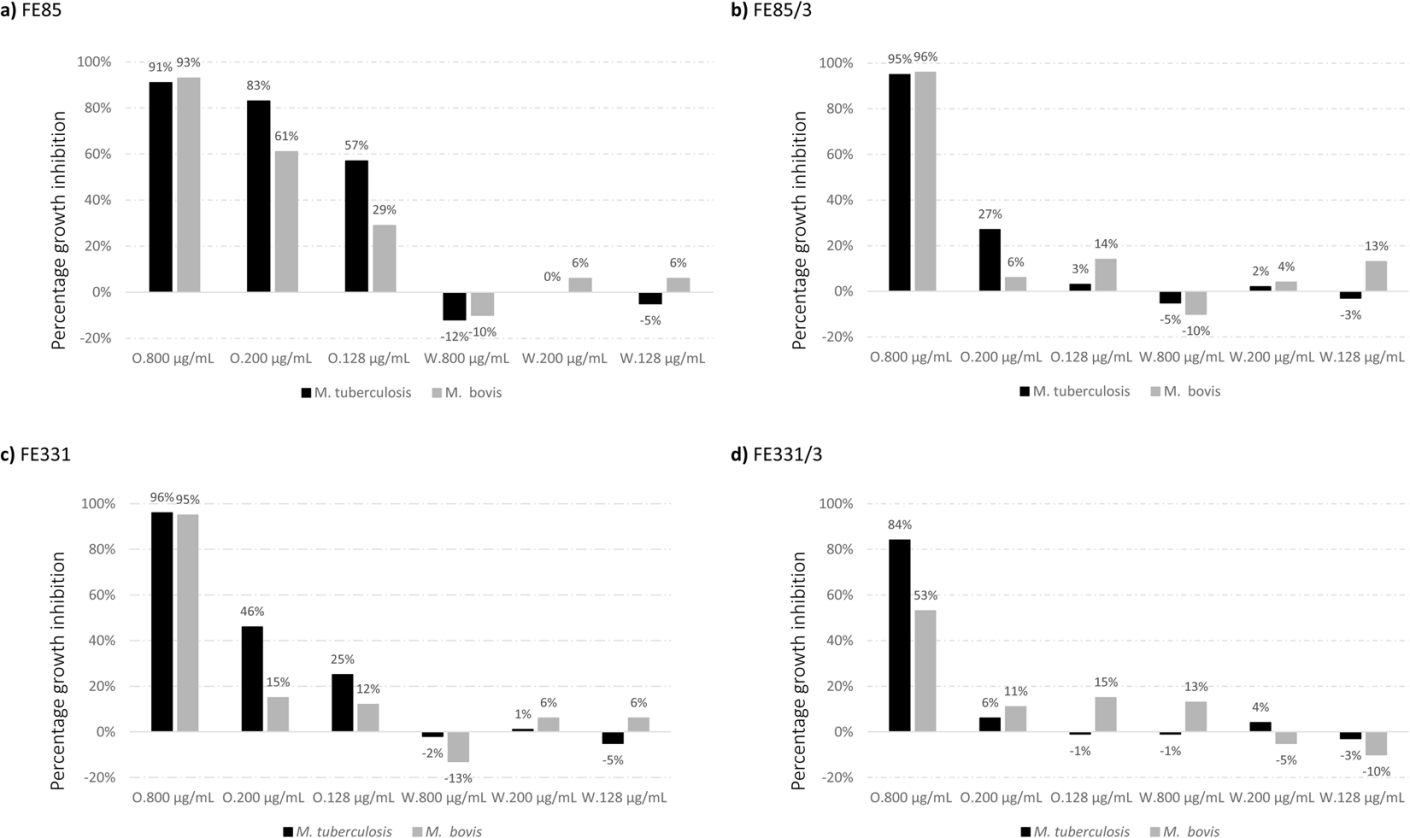
Percentage inhibition activity of *Skeletonema costatum* and *Chaetoceros pseudocurvisetus* by using the acetone/chloroform extraction method. Figure 3 reports the percentage inhibition activity of organic (O) and water (W) extracts of *Skeletonema costatum* FE85 and *Chaetoceros pseudocurvisetus* FE331 cultured in control and phosphate-starvation conditions (/1 and /3, respectively). Samples were extracted by using the acetone/chloroform method and tested at 800, 200 and 128 μg/mL.

Regarding the extracts obtained with the extraction method 2 (Acetone/Chloroform), both water and organic extracts were screened and at three different concentrations (800, 200 and 128 μg/mL). With this second extraction method, activity was found only for the organic extracts for both control and phosphate starvation conditions (Fig. 3). In particular, organic extracts of FE85 showed a concentration dependent inhibition of both *M*. *tuberculosis* and *M*. *bovis* (Student’s t-test, p < 0.001 for extracts of FE85 tested at 800 and 200 μg/mL against both *M*. *tuberculosis* and *M*. *bovis*; Fig. 3a), while extracts of FE85/3 were active only at the highest concentration (Student’s t-test, p < 0.001 for extracts of FE85/3 tested at 800 μg/mL against both *M*. *tuberculosis* and *M*. *bovis*, Fig. 3b).

Extracts of FE331 significantly reduced the growth of both *M*. *tuberculosis* and *M*. *bovis* at the highest concentration (Student’s t-test, p < 0.001 for all, except for FE331/3 against *M*. *bovis*), while at lower concentrations the activity was lost (Fig. 3c and c). Interestingly, the other two *S*. *costatum* strains screened in this study did not show activity, confirming that bioactivity is not only related to growth and stress conditions, but is also strain-dependent^11,12^.

Similarly we screened two strains of *Alexandrium andersonii*, two of *Asterionellopsis glacialis*, two of *Chaetoceros curvisetus*, two of *Chaetoceros socialis*, five of *Thalassiosira rotula* and nine of *Skeletonema marinoi*, but they were not active. Lauritano *et al*.^12^ found two *Skeletonema marinoi* strains active against *S. aureus*, FE60 and FE6, that inhibited *S. aureus* growth by 97% and 96%, respectively (Student’s t-test, p < 0.001). In particular, FE60 in nitrogen-starved and FE6 in phosphate-starved conditions. For this reason we selected and screened another 9 strains of this species to look for other possible bioactive strains. However, none of the new strains showed any activity (Student’s t-test, p > 0.05 for all). FE85 and FE331 were not active against the other microbes screened indicating a specific anti-tuberculosis activity. All the other microalgae tested in this study were not active against tuberculosis or other bacteria/fungus tested.

Cytotoxicity was analysed on human hepatocellular liver carcinoma (HepG2) cells to evaluate the potential toxic effect of the extracts. Hepatocytes were chosen because they are good models for studying toxicity since the liver is the primary site for drug metabolism and biotransformation^41,42^. None of the active extracts altered hepatocyte HepG2 cell survival (Student’s t-test, p > 0.05). Percentage of survival was 96% after 24 h exposure with FE331/1, 100% for FE331/2 and 98% for FE331/3. Similarly, percentage survival was 98% after 24 h exposure with FE85/1, 96% for FE85/2 and 98% for FE85/3. These results show that the active species did not show general cytotoxicity and can be considered for further development.

### Dereplication results

Since isolation and characterisation of new compounds is a very time consuming and costly step^43^, an early and quick dereplication by LC-UV-HRMS was performed to eliminate already known components using the platform available at MEDINA^40^. Our results clearly showed that extracts obtained with different extraction method procedures had different bioactivities. The anti-tuberculosis activity was confirmed by both extraction methods, but only the Amberlite resin was able to lead us to fractions with specific activity against *M*. *tuberculosis* or *M*. *bovis*. For this reason, bioactive extracts selected for dereplication were FE85/3 and FE331/3, obtained by chemical extraction Method 1 (i.e. with Amberlite resin).

**Figure 4:**
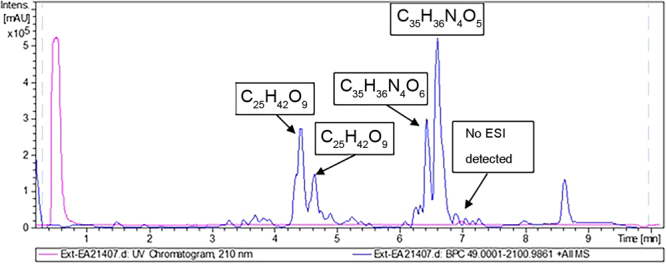
CHEMFE85/3 LC-UV trace showing the molecular formulae of the major components as determined by HRMS.

For FE85/3, the most abundant peaks found in the extract were (Fig. 4):

1. A peak with m/z of 609.2700. Dereplication using the dictionary of marine natural product database (http://dmnp.chemnetbase.com; DNP) identified 10-hydroxyphaeophorbide A as the most probable hit for this molecule. This is a known compound with a molecular formula of C_35_H_36_N_4_O_6_, molecular weight of 608.693 and an accurate mass of 608.263486. 10-hydroxyphaeophorbide A was previously isolated from the plants *Clerodendrum calamitosum* and *Neptunia oleracea*, the microalgae *Chlorella* sp. and from the tunicate *Trididemnum solidum*. The compound is known to have antioxidant, anti-inflammatory and anticancer activity^44^. It is a product of chlorophyll breakdown.
2. The second compound identified in these extracts was phaeophorbide A. This is also a known molecule that has been previously extracted from the seaweed *Grateloupia ellittica*^45^ and from the brown algae *Saccharina japonica*^46^. It has a molecular formula of C_35_H_36_N_4_O_5_, molecular weight of 592.693 and accurate mass of 592.268571. In addition, this compound is known to have the same properties as 10-hydroxyphaeophorbide A^45,46^.

**Figure 5:**
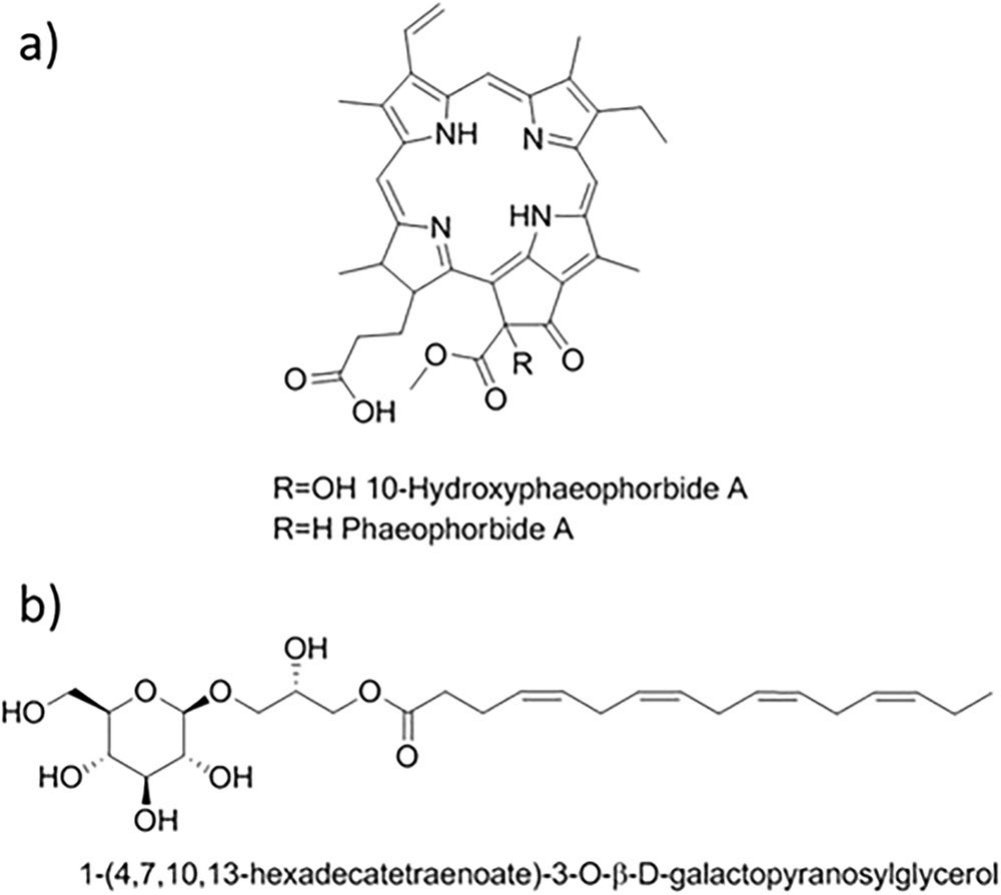
Structures of the major compounds identified in the active extracts by HRMS. 10-Hydroxyphaeophorbide A and Phaeophorbide A (**a**) and 1-(4,7,10,13-hexadecatetraenoate)-3-O-β-D-galactopyranosylglycerol (**b**) structures.

The 10-hydroxyphaeophorbide A and phaeophorbide A molecular structures are reported in Fig. 5a. Apart from these two molecules, the extract of FE85/3 also contained two intense peaks with molecular formulae of C_25_H_40_O_9_ and C_25_H_42_O_9_. The first of these compounds was tentatively identified as 1-(4,7,10,13-hexadecatetraenoate)-3-O-*β*-D-galactopyranosylglycerol (Fig. 5b) previously described in the DNP as a constituent of the marine alga *Oltmannsiellopsis unicellularis* NIES-359. The second is most probably a structurally related component lacking one of the double bonds, due to the similarity between the molecular formulae of both compounds. Finally, the main UV peak did not ionize under the analytical conditions tested. The retention time is over the chromatographic gradient and in the zone of fatty acids elution, indicating perhaps that this is the chemical nature of that component.

**Figure 6:**
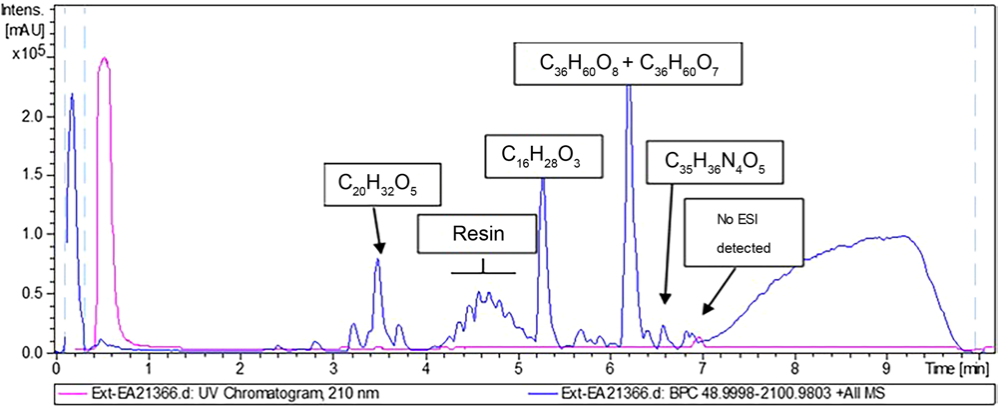
CHEMFE331/3 LC-UV-HRMS trace showing the molecular formulae of the major components as determined by HRMS.

Regarding FE331/3 (Fig. 6), apart from phaeophorbide A (C_35_H_36_N_4_O_5_), the extract also contained compounds with molecular formulae of C_36_H_60_O_8_ and C_36_H_60_O_7_, mainly identified previously as triterpene glycosides, C_16_H_28_O_3_, molecular formula not associated to any bioactive component in the DNP, and C_20_H_32_O_5_, identified as a medium component. As in the previous extract, the main UV peak did not ionize under the analytical conditions tested, but could be tentatively identified as a fatty acid component based on its retention time.

In conclusion, these preliminary analyses identified putative molecules that may be responsible for the anti-tuberculosis activity observed in this study. Further dereplication and/or successive purification and testing of the pure compounds will definitely associate the bioactivity detected in the original extracts with the presence of these or other molecules in the bioactive fractions.

## Conclusions

Our study is one of the few to test a wide range of marine microalgal species against *M*. *tuberculosis* and *M*. *bovis*. *S*. *costatum* (FE85) and *C*. *pseudocurvisetus* (FE331) are the first diatoms found to have anti-tuberculosis activity. The data indicated strain-, culturing condition- and extraction method-related differences in the biological activity.

When the culturing parameters are modified, the same organism may show different bioactivities and produce diverse compounds^47^. This strategy, termed OSMAC (One strain–many compounds), has successfully been applied for drug discovery in bacteria^47^ and could also work in microalgae. The production of primary and secondary metabolites in microalgae can vary depending on several factors^48^, such as growth phases^49^, strains^50^, light^51^, temperature^52^, culturing media^53^, grazing pressure^54^ and extraction method^55^. This metabolic plasticity positively influences bioactivities and hopefully leads to the discovery of novel bioactive compounds for the treatment of human diseases.

Extracts obtained by different extraction methods clearly showed bioactivity differences in this study. However, this may have been expected considering that the acetone/chloroform extraction is a gross procedure, while the Amberlite resin favours the extraction of specific components, i.e. hydrophobic compounds up to 40,000 MW (Sigma-Aldrich). Amberlite XAD16N extraction might not be retaining all the compounds present in the diatoms, leaving some polar unextracted components in the water, whereas the extraction with organic solvent, in which both, the organic and aqueous phases are dried and tested, provides a more comprehensive set of all the metabolites present in the extracts, explaining the different results obtained in terms of bioactivity. Early LC-UV-HRMS dereplication tentatively identified some components that might explain the bioactivity observed, but confirmation of this hypothesis will require the isolation and biological testing of individual compounds in the extract. Future work in this area will therefore require the preparation of scaled-up cultures and fractionation using a combination of chromatographic approaches.
