# Author Correction: First identification of marine diatoms with anti-tuberculosis activity

**DOI:** 10.1038/s41598-018-24176-7

**Published:** 2018-04-17

**Authors:** Chiara Lauritano, Jesús Martín, Mercedes de la Cruz, Fernando Reyes, Giovanna Romano, Adrianna Ianora

**Affiliations:** 1 Stazione Zoologica Anton Dohrn, Department of Integrative Marine Ecology, Naples, Italy; 2 Fundación MEDINA, Centro de Excelencia en Investigación de Medicamentos Innovadores en, Andalucía, Avda. del Conocimiento 34, Granada, 18016 Spain

Correction to: *Scientific Reports* 10.1038/s41598-018-20611-x, published online 02 February 2018

The original version of this Article contained errors in the Abstract.

“Results showed that extracts of two diatoms, *Skeletonema tropicum* and *Chaetoceros pseudocurvisetus*, had anti-tuberculosis activity and were active only when cultured in the control and phosphate-starvation conditions, while the nitrogen starvation condition showed no activity”.

now reads:

“Results showed that extracts of two diatoms, *Skeletonema costatum* and *Chaetoceros pseudocurvisetus*, had anti-tuberculosis activity and were active only when cultured in the control and phosphate-starvation conditions, while the nitrogen starvation condition showed no activity”.

These errors have now been corrected in the PDF and HTML versions of the Article.

